# A Dual-Modality Hybrid Imaging System Harnesses Radioluminescence and Sound to Reveal Molecular Pathology of Atherosclerotic Plaques

**DOI:** 10.1038/s41598-018-26696-8

**Published:** 2018-06-12

**Authors:** Raiyan T. Zaman, Siavash Yousefi, Steven R. Long, Toshinobu Saito, Michael Mandella, Zhen Qiu, Ruimin Chen, Christopher H. Contag, Sanjiv S. Gambhir, Frederick T. Chin, Butras T. Khuri-Yakub, Michael V. McConnell, K. Kirk Shung, Lei Xing

**Affiliations:** 1 Division of Cardiovascular Medicine, Department of Medicine, Stanford University School of Medicine, Stanford, USA; 2 Division of Medical Physics, Department of Radiation Oncology, Stanford University School of Medicine, Stanford, USA; 3 Department of Pathology, Stanford University School of Medicine, Stanford, USA; 4 Department of Pediatrics (Neonatology), Stanford University School of Medicine, Stanford, USA; 5 Department of Radiology, Stanford University School of Medicine, Stanford, USA; 6 Department of Biomedical Engineering, Viterbi School of Engineering, University of Southern California, Stanford, USA; 7 Molecular Imaging Program at Stanford University (MIPS), Stanford University School of Medicine, Stanford, USA; 8 Department of Microbiology and Immunology, Stanford University School of Medicine, Stanford, USA; 9 Department of Bioengineering, Stanford University Schools of Medicine and of Engineering, Stanford, USA; 10 Department of Electrical Engineering, Stanford University, Stanford, USA; 11 Department of Radiology, Harvard medical School, Boston, MA 02115 USA; 12 Massachusetts General Hospital 149 13th Street, Room 5406 Charlestown, Massachusetts, 02129 USA; 13 Present Address: Michigan State University, Michigan, USA

## Abstract

Atherosclerosis is a progressive inflammatory condition caused by an unstable lesion, called thin-cap fibro atheromata (TCFA) that underlies coronary artery disease (CAD)—one of the leading causes of death worldwide. Therefore, early clinical diagnosis and effective risk stratification is important for CAD management as well as preventing progression to catastrophic events. However, early detection could be difficult due to their small size, motion, obscuring ^18^F-FDG uptake by adjacent myocardium, and complex morphological/biological features. To overcome these limitations, we developed a catheter-based **C**ircumferential-**I**ntravascular-**R**adioluminescence-**P**hotoacoustic-**I**maging (CIRPI) system that can detect vulnerable plaques in coronary arteries and characterizes them with respect to pathology and biology. Our CIRPI system combined two imaging modalities: **C**ircumferential **R**adioluminescence **I**maging (CRI) and **P**hoto**A**coustic **T**omography (PAT) within a novel optical probe. The probe’s CaF_2_:Eu based scintillating imaging window provides a 360° view of human (n = 7) and murine carotid (n = 10) arterial plaques by converting β-particles into visible photons during ^18^F-FDG decay. A 60× and 63× higher radioluminescent signals were detected from the human and murine plaque inflammations, respectively, compared to the control. The system’s photoacoustic imaging provided a comprehensive analysis of the plaque compositions and its morphologic information. These results were further verified with IVIS-200, immunohistochemical analysis, and autoradiography.

## Introduction

Coronary artery disease (CAD) is one of the leading causes of morbidity and mortality worldwide. About 60–70% of acute coronary syndromes are due to an unstable lesion called thin-cap-fibro-atheromata (TCFA), a slow progressive condition. Despite its high prevalence, it is difficult to image coronary plaque activities due to its relatively small size, motion, obscuring signal from adjacent myocardium, and complex morphological and biological features. Therefore, early clinical diagnosis of TCFA or vulnerable plaques, is critical in preventing catastrophic events such as myocardial infarction (heart attack) and ultimately death. To address this challenge, various intravascular imaging modalities such as intravascular ultrasound (IVUS), optical coherence tomography (OCT), fluorescence, and photoacoustic imaging^1–7^ have been developed for detecting plaques in coronary arteries. Although, these tools provide structural characteristics of the imaged vessel, they are limited in characterizing the biological aspects of the disease^8,9^ such as inflammation or angiogenesis^10–12^.

There is an unmet need in cardiovascular research to predict the evolution of coronary atherosclerosis and identify culprit lesions that are likely to rupture. Multiple molecular imaging agents are conjunction with several imaging modalities yield the detection and characterization of atherosclerotic plaques in large vessels^12^. For example, iodinated nanoparticulate contrast agent, N1177, for computed tomography (CT)^13^; ^18^F-FDG for positron emission tomography (PET)^2^; ultra-small super-paramagnetic iron oxide probes for magnetic resonance imaging (MRI)^14,15^; and VCAM-1-specific ^99m^Tc-labeled peptidic sequences for single photon emission computed tomography (SPECT)^16^. Despite the importance of this information, these conventional imaging modalities with molecular probes have lacked the resolution or sensitivity for accurately detect coronary vulnerable plaque. Information on atheroma burden, its microstructure, and its cellular and molecular composition may dramatically affect prognosis. There is evidence that acute coronary events occurrence and their outcomes depend on both the austerity of luminal narrowness with inflammation and on the characteristics of plaque morphology^17^. Despite considerable effort has been made to understand the effects of local and systemic factors on plaque progression by visualizing the coronary anatomy and pathology, the sensitivity and specificity of imaging modalities remains inadequate^18^.

To enable detection and characterization of vulnerable coronary plaques, we designed, developed, and tested a hybrid catheter-based CIRPI system with the intent of providing effective detection and biomolecular characterization of coronary TCFA by optically capturing radionuclide information on vulnerable plaque biology (e.g., ^18^F-FDG uptake) combined with photoacoustic imaging for morphologic classification of vulnerable plaque compositions (e.g., cholesteryl ester, phospholipids)^19^. This method was designed to address the current limitations of TCFA detection and enable a more detailed study of the plaque structural, biological, and morphological components. A catheter-based system allows higher resolution and sensitivity, plus is more immune to motion. The dual-modality hybrid imaging approach can provide a detailed and comprehensive assessment of vulnerable plaques not available from other currently available imaging modalities. We imaged both human and murine *ex vivo* carotid plaques with histologic validation and demonstrate that CIRPI permitted high sensitivity plus detailed tissue characterization of vulnerable plaque pathobiology.

## Materials and Methods

### Circumferential Intravascular Radioluminescence-Photoacoustic Imaging (CIRPI)

**Figure 1:**
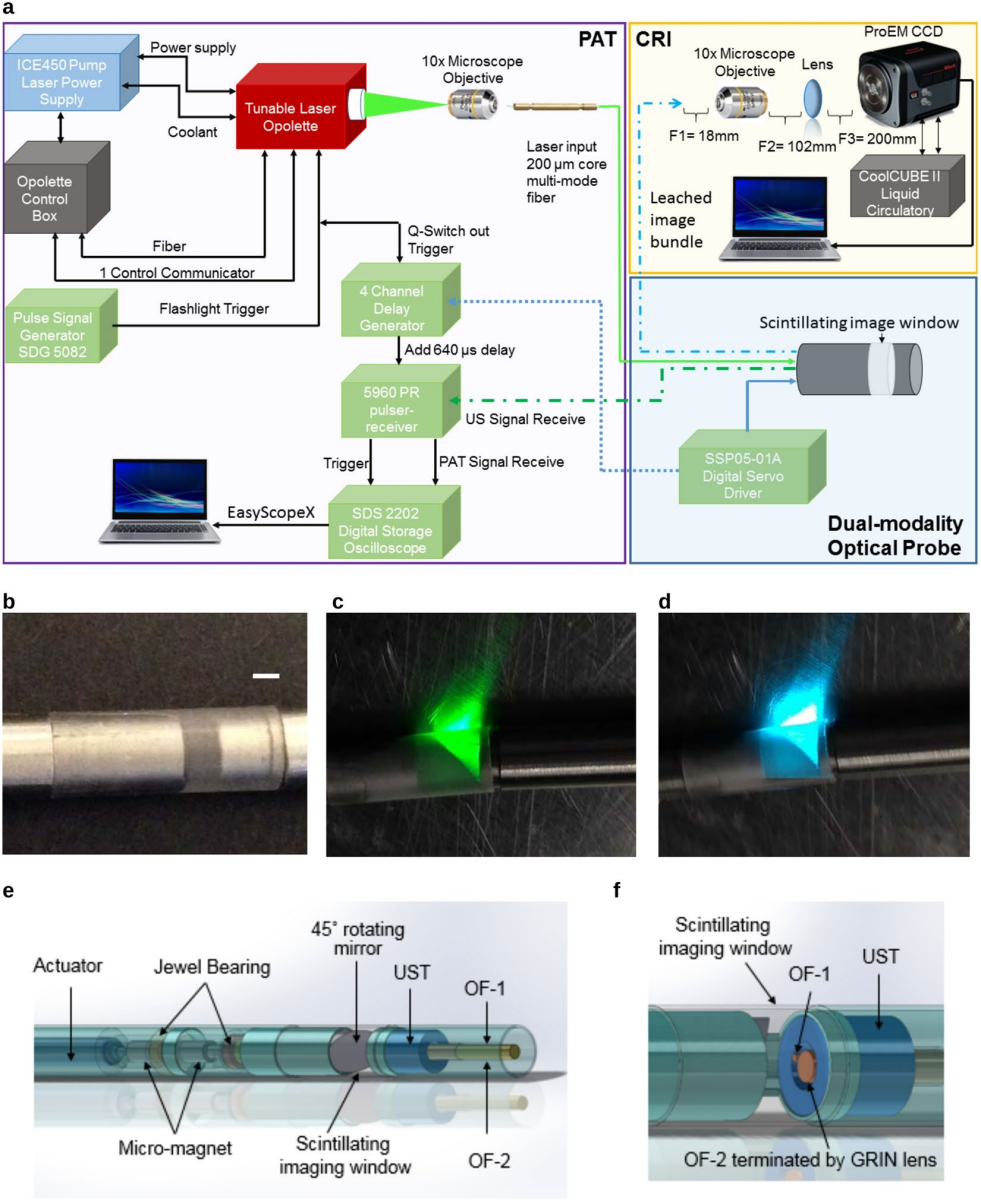
Schematic diagram of **(a)** the dual-modality CIRPI system for detection and characterization of atherosclerotic plaque. The CIRPI system has three main components (1) CRI, detects and outlines the location of vulnerable plaque by identifying macrophage accumulations, (2) PAT, characterize the plaque by disease tissue compositions, and (3) optical probe that collects radioluminescent and PA signals. CRI peripheral system consists of (1) a 10× magnification infinity-corrected microscope objective (2) an infinity-corrected tube lens for plan fluoride objective in between the objective (F2 = 102 mm) and the ProEM charge-coupled device (CCD) camera (F3 = 200 mm). PAT system consists of (1) tunable laser, (2) pulse signal generator (3) 4-channell delay generator (4) pulser-receiver (5) oscilloscope (6) laptop with EasyScopeX software. **(b)** Photograph of the novel probe with scintillating imaging window made from CaF_2_:Eu phosphor (scale bar: 1 mm), CaF_2_:Eu converts beta particles to visible photons **(c)** tunable laser light is delivered through water coupled scintillating window at 540 nm (Visible, green), 560 nm (Visible, green), and 1040 nm wavelength (NIR, Near InfraRed) **(d)** at 1180, 1210, and 1235 nm wavelength (IR, InfraRed); at 920 nm wavelength (NIR, not shown). **(e)** Schematic diagram of the novel probe design shows the main components (1) CaF_2_:Eu scintillating imaging window, (2) OF-1: 0.2 mm core multimode light guiding optical fiber, (3) OF-2: 18 K pixels imaging fiber, (4) UST: single element unfocused ultrasonic transducer, (5) digital actuator, and (6) 45° degree flat rotating mirror **(f)** enlarged image of the imaging window illustrates the orientation of OF-1, OF-2, and UST. Figure 1 is already published in the SNMMI Annual Conference Abstract^19^.

See Supplementary sections and Fig. 1 for the detail descriptions of this CIRPI system. Figure 1 is already published in the SNMMI Annual Conference Abstract^19^.

### Verification Imaging

See Supplementary sections.

### Statistical Analysis

See Supplementary sections.

### Experimental Procedures

#### *Ex Vivo* imaging of Murine Plaque Uptake of ^18^F-FDG

All murine experiments in this study were conducted based on an approved protocol by the Stanford Administrative Panel on Laboratory Animal Care (APLAC 9941). We developed macrophage-rich atherosclerotic lesions in the left carotid artery (LCA) of diabetic, hyperlipidemic FVB/NJ murine model (n = 10), as studied extensively in our laboratory^20–23^. In brief, a high-fat diet containing 40% kcal fat, 1.25% (by weight) cholesterol, and 0.5% (by weight) sodium cholate (D12109, Research Diets, Inc., New Brunswick, NJ, USA) were fed to 8-week-old male murine models. After one month on this diet, diabetes was induced by daily intraperitoneal injections of streptozotocin (STZ, 40 mg/kg, Sigma-Aldrich) for 5 consecutive days. Two weeks after the diabetes initiation, a 5-0 silk (Ethicon) ligature was used to ligate the LCA below the bifurcation while the right carotid artery (RCA, negative control) was kept intact. The procedure was performed with 2% Isoflurane.

**Figure 2:**
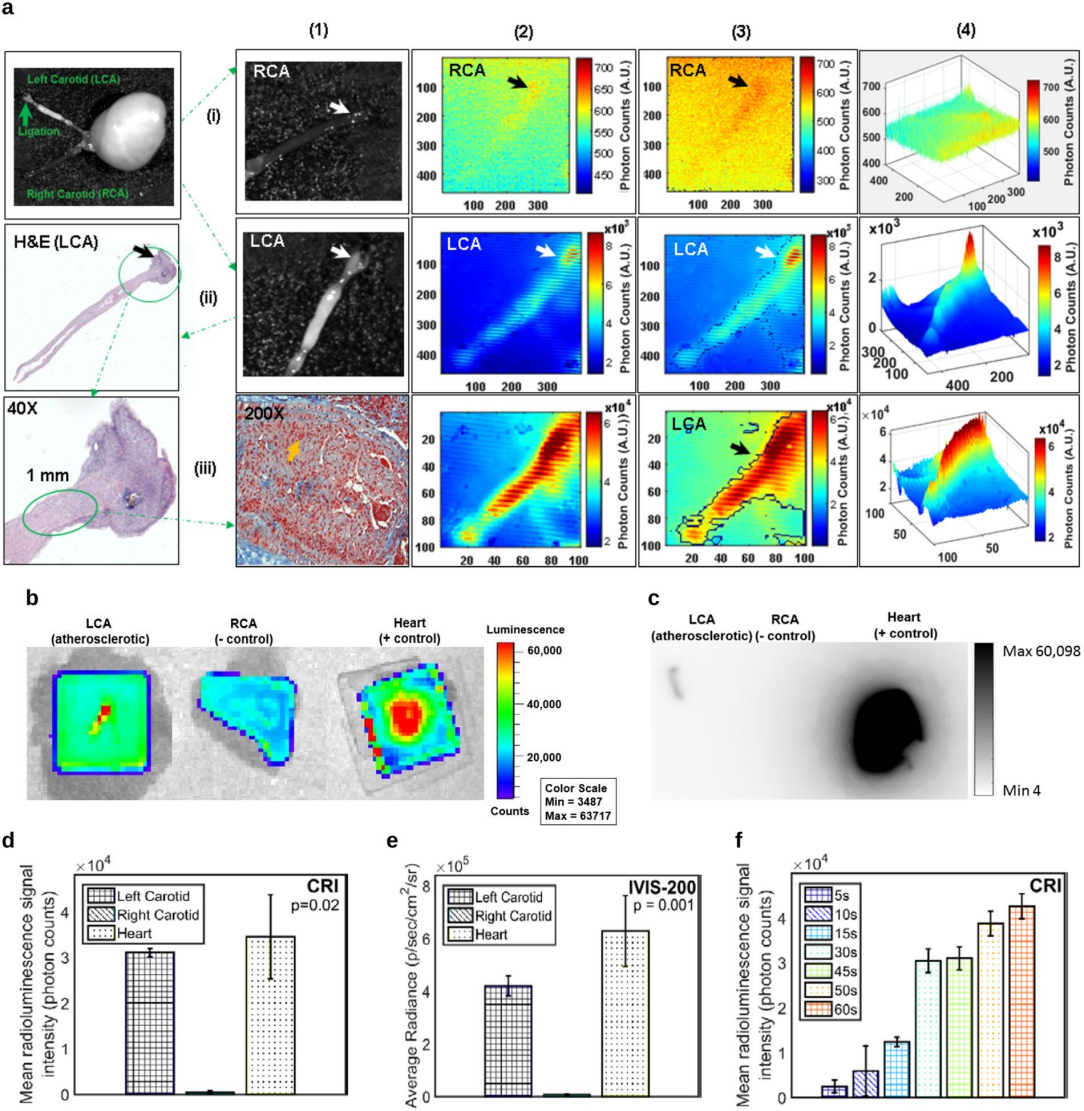
Murine carotid artery images reveal pathognomonic features of plaques. **(a)** CRi Maestro images of *ex vivo* murine carotid arteries are taken one hour after 200 µCi of ^18^F-FDG IV injection, (i-1) non-ligated RCA (negative control, arrow points to the location where the edge detection software is implemented) and (ii-1) ligated LCA (arrow points to the ligation location, enlarged bulb shaped area, due to macrophage accumulation; long white area below the arrow is an artifact due to reflected light from CRi Maestro imaging system), and (iii-1) a 10× magnification of the full length histologic image of the LCA. After a 200× magnification, the dilated area of LCA near the ligation shows macrophage accumulation nearly filling the lumen of the vessel (yellow arrow) that is extend to 1 mm from the ligation location. The CRI images of (i-2) RCA shows only 700 photon counts, a trace amount of radioluminescent signal, where (ii-2) LCA shows almost 8 × 10^3^ photons at the ligation and its surrounding area representing the presence of larger number of macrophages within the atherosclerotic plaque (1 × 1 binning). (iii-3) However, when the binning is increased to 4 × 4, the signal intensity rises to 60 × 10^3^ photon counts and the radioluminescent signal extends to 1 mm from the ligation. (i-3) A custom written edge detection software is unable to show any edges in RCA, a clear indication of absence of macrophages (ii-3, iii-3) LCA highlights the area of macrophages with prominent edges. X and Y axes in these images (i-2, ii-2, iii-3, i-3, ii-3, iii-3) represent the effective active imaging resolutions in pixels. (i-4) The contour plot of RCA highlights a flat radioluminescent signal distribution and (ii-4, iii-4) a sharp peak is observed at the LCA plaque area. Confirmatory imaging with **(b)** IVIS-200 system (after a LSO scintillating screen is placed on the top of each sample) and **(c)** autoradiography show similar results as the CIRPI system. Statistical plots show that **(d)** the CIRPI images of LCAs are 63-fold brighter compared to RCAs (45 second exposure time) where **(e)** the IVIS-200 images of the same LCAs are 65-fold brighter than the RCAs (45 second exposure time), **(f)** the CIRPI images of all murine LCAs show a linear relationship between the radioluminescent signal intensity vs. exposure time. Radioluminescence is produced within the scintillating imaging window following the emission of a beta particle from a radiotracer (^18^F-FDG) within a macrophage. The optical photons were captured by a high-numerical-aperture 10× microscope objective coupled to a deep-cooled ProEM CCD camera. Radioluminescence signal was measured as photon counts in arbitrary units (A.U.). All results statistically significant (P < 0.05). Figure 2 is already published in the SNMMI Annual Conference Abstract^19^.

Two weeks after the ligation surgery, murine models were fasted for 6 hours before 200 μCi of ^18^F-FDG (0.5 CC injection volume with a 28$1/2$G hypodermic needle) was administered intravenously (IV). One hour after IV injection, the ligated LCA and non-ligated RCA, and heart (positive control) were harvested to image intact with the CRi Maestro fluorescence imaging system (Fig. 2a:i-1,ii-1). Then the tissues were placed individually (without being cut open longitudinally) under a Lutetium Oxyorthosilicate (LSO) scintillating crystal screen to enable imaging with the IVIS-200 system for primary verification of glucose uptake by the inflamed plaque (Fig. 2b). The ^18^F-FDG uptake within the atherosclerotic plaque was then imaged with our CIRPI system when the probe was placed in close contact with the tissue to capture all deposited energy from β-particles during ^18^F-FDG decay. The exposure time for the CIRPI system was chosen to be 1, 5, 10, 15, 30, 45, 50, and 60 seconds to achieve adequate signal intensity (the 45 second exposure was most informative and is the only data shown and discussed). All murine samples (n = 10) were then imaged for photoacoustic signal with our CIRPI system for plaque characterization by identification of plaque morphological constituents, then verified with Vevo LAZR/Vevo 2100. Of the 10 total plaques, 6 were then co-registered with histochemical analyses and 4 were used for autoradiography (Fig. 2c), as an additional verification of the radioluminescence images taken with our CIRPI system.

#### *Ex Vivo* imaging of Human Carotid Plaque Uptake of ^18^F-FDG

Approximately 25–28 mm length plaques were excised from living individuals obtained at carotid endarterectomy surgery (n = 6, five males and one female patients ages between 63–82 years); we did not impose restrictions on age and included men and women in our protocol. This study on human endarterectomy samples was conducted according to an approved Institutional Review Board (IRB) protocol by Stanford University Research Compliance Office (IRB 22141). However, as carotid endarterectomy sample is considered as surgical waste, it does not meet Federal definition of research or clinical investigation and requires no consent from patients prior to surgery. Carotid specimens were initially placed in phosphate buffered saline (PBS) on ice during transportation, then warmed naturally to room temperature for experiments. The time between surgical extraction of carotid plaque, local injection of ^18^F-FDG, and measurements was ~1 h. The carotid specimen was cut opened longitudinally and placed lumen side up on a white taped petri dish to minimize light absorption. The injection site of ^18^F-FDG was selected randomly as the entire human tissue sample was plaque. Several measurement points were chosen from diseased regions, which were then imaged with our CIRPI system pre- and post-injection of a single 50 µCi of ^18^F-FDG (0.25 CC injection volume with a 281/2G hypodermic needle). The scintillating imaging window of the CIRPI probe was then placed in direct contact with the tissue samples near the injection site to capture all deposited energy from β-particles during the ^18^F-FDG decay. The exposure time for the CIRPI system was same as in the murine model. These findings with the CRI peripheral system were verified using the IVIS-200 to image light after a LSO crystal was placed on top of the injection site. The same plaque was then imaged with our PAT peripheral system for characterization of morphologic constituents and verified with Vevo LAZR/Vevo 2100. All carotid samples underwent histochemical analysis.

### Data Availability Statement

All the generated data in this study were analyzed and included in this manuscript in form of Figures and Table.

## Results

### Murine Carotid Artery

#### Circumferential Radioluminescence Imaging (CRI)

Our murine carotid-ligation model develops localized, inflamed plaques. The CIRPI probe had high sensitivity for detecting ^18^F-FDG uptake in the inflamed regions of the murine LCA (Fig. 2a:ii-1), compared to the negative control, RCA (Fig. 2a:i-1). Quantitatively, CIRPI (Fig. 2d) detected a 63-fold higher radioluminescent signal through CRI from macrophage-rich atherosclerotic plaques in ligated LCA compared to RCA (3.10 × 10^4^ ± 9.41 × 10^2^ vs. 4.90 × 10^2^ ± 2.40 × 10^2^ photon counts, p = 0.02) using a 45 second exposure time. Our edge detection software outlined the areas of high radioluminescent signal intensity in the LCA with high sensitivity (Fig. 2a:ii-3) and showed no edges in the RCA (Fig. 2a:i-3). The contour plots of LCA (Fig. 2a:ii-4) highlighted higher signal distribution associated with the ligated regions in comparison to rest of the LCA tissue; RCA showed flat signal distribution at all exposure times (Fig. 2a:i-4). When binning was increased to 4 × 4, we observed higher radioluminescence signal extended 1 mm beyond the ligation location (Fig. 2a:iii-2, iii-3, iii-4). This finding was further confirmed and co-registered with the histology study (2a:iii-1), where we observed 1.0 mm of macrophage accumulation beyond the ligation location (green oval). Confirmatory results were obtained using a Lutetium Oxyorthosilicate (LSO) scintillating crystal screen and an IVIS-200 optical imaging system (Fig. 2b), and these images exhibited a similar average of a 65-fold brighter radioluminescent signal (Fig. 2e) from LCA compared to RCA (4.20 × 10^5^ ± 3.83 × 10^4^ vs. 6.43 × 10^3^ ± 2.71 × 10^3^ p/sec/cm^2^/sr, p = 0.001). The correlation of CIRPI and IVIS-200 results were statistically significant (P < 0.05). A linear relationship was observed between the average radioluminescent signal intensity from all LCA vs. exposure times (Fig. 2f). Also, high radioluminescent signals from ^18^F-FDG were detected in the heart. As further confirmation, these results with our CIRPI system were co-registered with autoradiography where LCA exhibited 51,289 photons at the ligation location and no signal was detected from RCA (Fig. 2c). Figure 2 is already published in the SNMMI Annual Conference Abstract^19^.

#### Photoacoustic Tomography (PAT) Imaging

**Figure 3:**
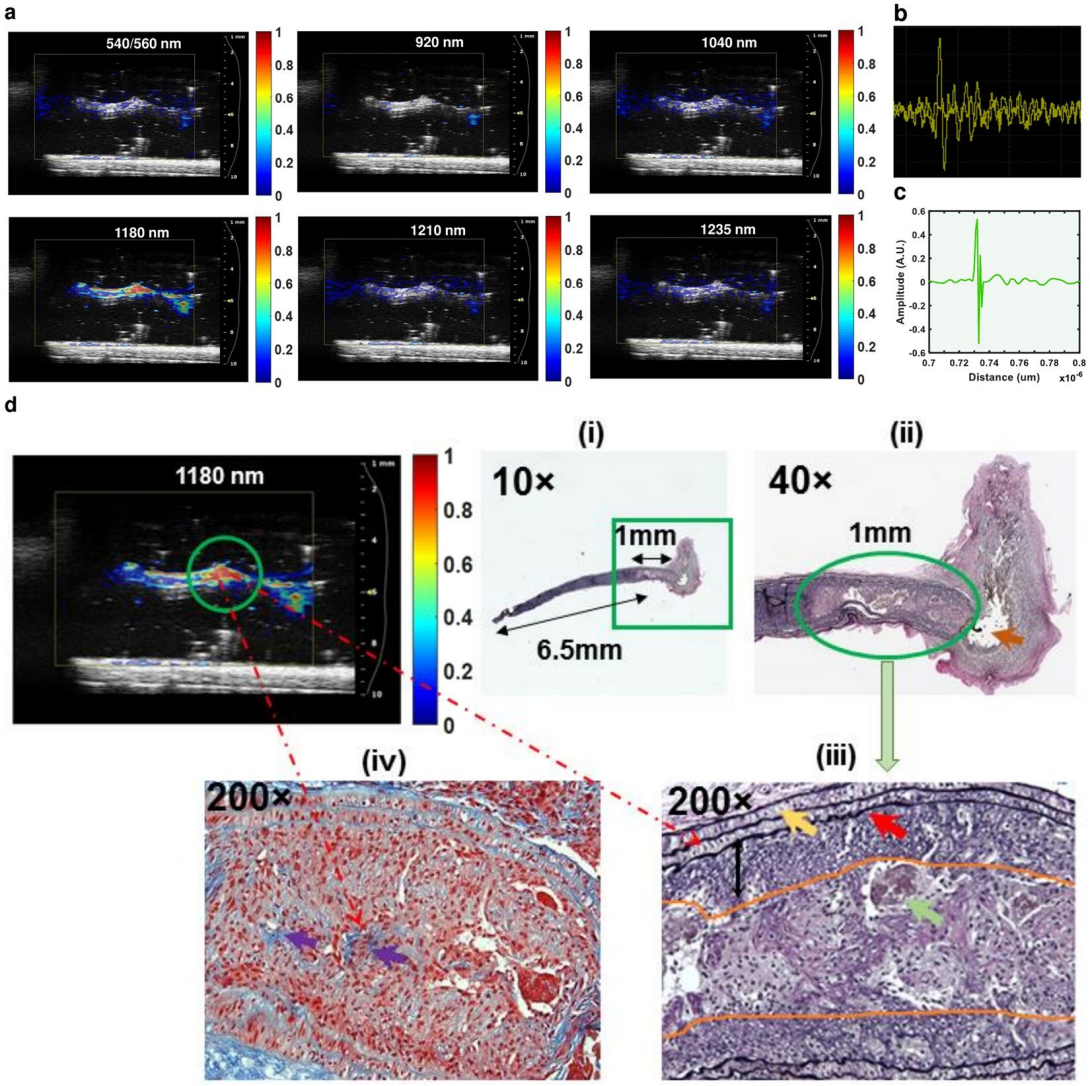
CIRPI images correlate with conventional ultrasound and histological images. **(a)** Reconstructed PAT images at multiple wavelengths are superimposed on a Vevo 2100 collected ultrasound image of the same murine LCA shown in Fig. 2. The PAT images show a precise alignment with the ultrasound images. For this specific murine model, there is no other plaque compositions identified using the CIRPI system except for elastin and collagen at 1180 nm wavelength representing an area of 7.5 mm in length. **(b)** A single PA signal (A-line) is captured with an oscilloscope; **(c)** an A-line is captured when the CIRPI probe is placed in close proximity of 0.8 µm and laser is excited at 1180 nm wavelength using a 7 ns tunable pulsed laser at 20 Hz repetition rate with 100% energy efficiency. Histochemical analysis at **(d-i)**10× magnification of this 7.5 mm long (without the bulb or ligation location) LCA sample (EVG stain) is further magnified to **(d-ii)** 40× to highlight the suture material at the ligation end (orange arrow) and the adjacent dilated 1 mm long vessel lumen (green oval), containing macrophage accumulation, thrombus and small amounts of collagen deposition. **(d-iii)** 200× magnification of the dilation area near the ligated end of the LCA shows thrombus (green arrow), black staining elastic fiber (red arrow), muscle fiber (yellow arrow), 0.5 mm thick muscle wall (black double arrow) and 1 mm long area of macrophage accumulation (orange highlight) located at 6.5 mm away from the non-ligated end of the LCA. **(d-iv)** Trichrome stain of dilatation area near the ligated end of LCA shows small strands of collagen highlighted in blue (purple arrow) with visible macrophage presence. According to the histochemical analyses, although there were extensive inflammation cells at the suture site as well as the perivascular area, these inflammatory cells provided no background signal for the PAT image of the murine LCA. All PA signals that were detected with our CIRPI system at 1180 nm was generated from the optical absorbers elastic fiber and collagen due to their thermo elastic expansion causing an acoustic pressure wave. Both EVG and trichrome stained sample illustrate one-to-one correlation to the highlighted area of the PAT image at 1180 nm wavelength associated with the CIRPI system (R^2^ = 0.97, p < 10^−5^). Figure 3 is already published in the SNMMI Annual Conference Abstract^19^.

Reconstructed PAT images based on murine LCA plaques over a range of wavelengths showed a prominent photoaccoustic (PA) signal at 1180 nm that elucidated the presence of elastin/collagen throughout the length of the vessel. This elastin/collagen aligned well with the Vevo 2100 ultrasound image of the same murine LCA when superimposed (Fig. 3a). No significant PA signals at other wavelengths were detected, implying no substantial regions of mineralization/calcium or lipid/fatty acid accumulation in the murine LCA.

#### Histologic Analysis

Review of the full-length LCA specimen showed areas of macrophage accumulation that was 1 mm in length and located 6.5 mm from the non-ligated end of the mouse artery (Fig. 3d-i). The tip of the LCA near the sutured end showed thrombi and macrophage accumulation that nearly filled the lumen of the vessel (Fig. 3d-ii). The EVG stain further investigated the dilation area near the ligated end of the LCA and showed thrombus (green arrow), black staining elastic fiber (red arrow), muscle fiber (yellow arrow), 0.5 mm thick muscle wall (black double arrow), and 1 mm length area macrophage accumulation (orange highlight) located 6.5 mm from the non-ligated end of the artery in the mouse (Fig. 3d-iii). Trichrome stain of the dilated area near the ligated end showed small strands of collagen (purple arrows) embedded within areas of significant macrophage accumulation as well as throughout the vessel wall with focal thrombus formation (Fig. 3d-iv). Figure 3 is already published in the SNMMI Annual Conference Abstract^19^. This quantitative histochemical analysis bolstered our CIRPI findings of macrophage accumulation (via CRI) and significant elastin/collagen (via PAT) in the murine LCA. The remaining portion of the murine LCA demonstrated mild sub-endothelial macrophage accumulation without significant inflammation, calcification or luminal narrowing/occlusion. Overall, in the histology study all LCA showed 10% or less lumen occlusion and presented a strong correlation with the CIRPI findings for elastin/collagen (R^2^ = 0.97, p < 10^−5^) and macrophages (R^2^ = 1 for, p < 10^−5^). See Table 1 in *Supplementary Results* for details on histochemical analysis and CIRPI results of murine model.

### Human Carotid Artery

#### CRI

**Figure 4:**
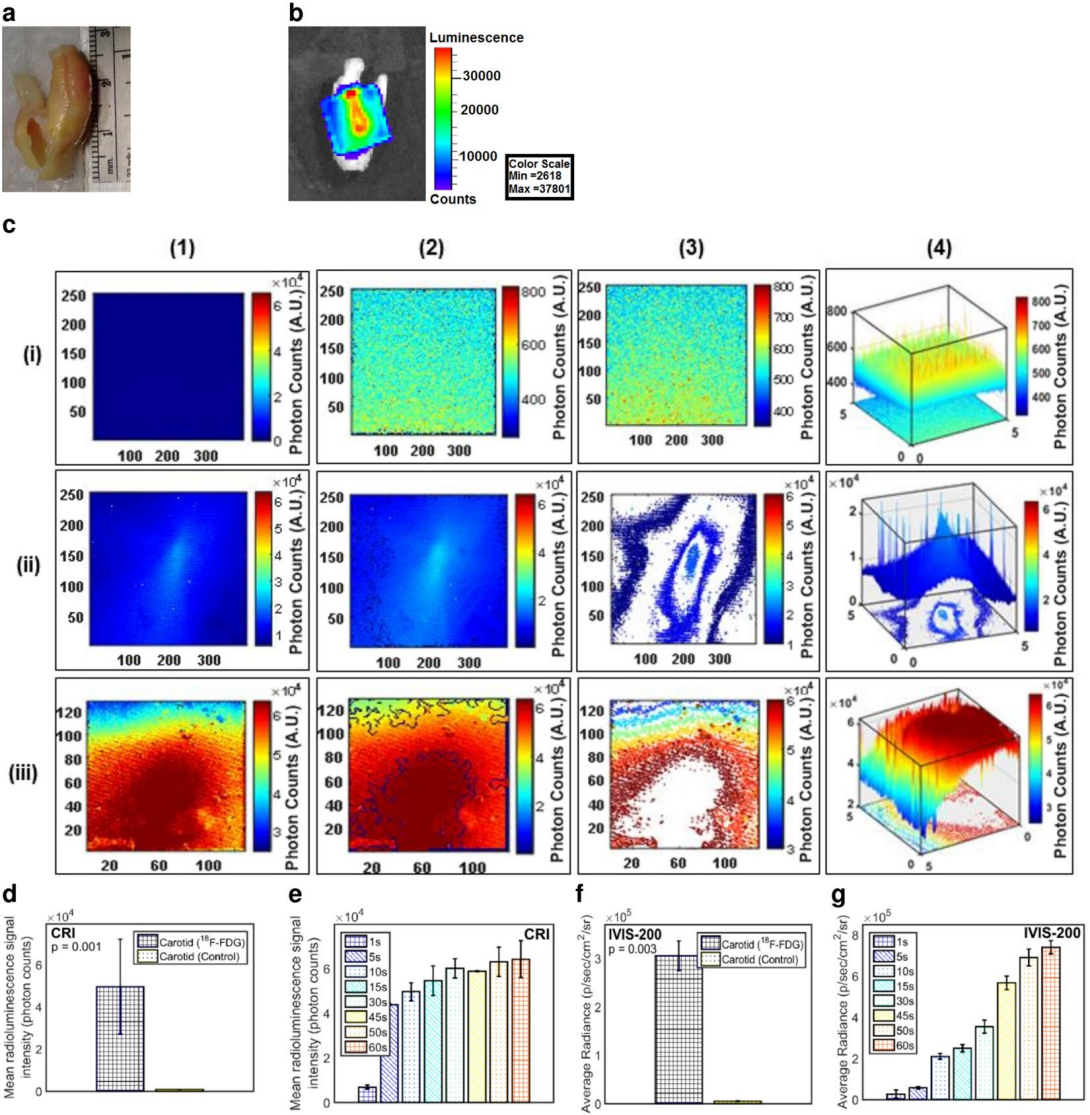
Human carotid endarterectomy sample images reveal pathognomonic features of plaques **(a)** photograph is taken with a smart phone, **(b)** a confirmatory IVIS-200 image yields high radioluminescent signal at the ^18^F-FDG injection site during the sample is placed under a scintillating LSO crystal screen (45 second exposure time at 8 × 8 binning). The sample is rotated to highlight the location of the ^18^F-FDG accumulation. **(c)** CRI image of (i-1) control sample (pre-injection) shows no radioluminescent signal with medium 4 × 4 binning; however, (ii-1) post-injection of 50 µCi ^18^F-FDG shows an immediate increase of radioluminescent signal with 2 × 10^4^ photons at small 1 × 1 binning, (iii-1) 6 × 10^4^ photons at medium 4 × 4 binning. A custom written edge detection software outlines the area with this high radioluminescent signal. (i-2) No edges are detected in the control, representing the absence of ^18^F-FDG. (ii-2, iii-2) Post-injection image shows a distinctive edge at the deposition of ^18^F-FDG. A custom written software highlights the contour of the ^18^F-FDG signal distribution. (i-3) No contour is detected in the control sample where (ii-3, iii-3) post-injection area of the atherosclerotic plaque showed well defined contour pattern. X and Y axes in these images (i-1, ii-1, iii-1, i-2, ii-2, iii-2, i-3, ii-3, iii-3) represent the effective active imaging resolutions in pixels. 3D plot of the radioluminescent signal intensity shows (i-4) a flat distribution of signal in the control, and (ii-4, iii-4) a sharp peak of radioluminescent signal at the post-injection area. Statistically based on CRI images of **(d)** all human carotid endarterectomy samples show a 60-fold higher radioluminescent signal at the atherosclerotic plaque area compared to the control (45 second exposure time with binning of 4 × 4); **(e)** a linear relationship is identified between radioluminescent signal intensity vs. exposure time. Similarly the confirmatory IVIS-200 images **(f)** yield a 62-fold brighter radioluminescent signal compared to the control (45 second exposure with binning of 4 × 4); **(g)** a linear relationship is established between radioluminescent signal intensity vs. exposure time. All results statistically significant (P < 0.05). Figure 4 is already published in the SNMMI Annual Conference Abstract^19^.

A representative photograph of a 28 mm long human carotid endarterectomy sample is shown in Fig. 4a. The CRIPI probe detected high radioluminescent signal from post-^18^F–FDG-injection carotid endarterectomy samples (Fig. 4c:ii-1, iii-1) compared to pre-injection control (Fig. 4c:i-1). Data showed a 60-fold higher CRIPI radioluminescent signal from the human carotid plaques post-injection vs. control (4.96 × 10^4^ ± 2.25 × 10^4^ vs. 8.37 × 10^2^ ± 6.63 × 10^1^ photon counts, p = 0.001, Fig. 4d). The edge detection software outlined areas of high radioluminescent signals at the site of ^18^F-FDG injection (Fig. 4c:ii-2, iii-2) unlike the control, which showed no defined edges (Fig. 4c:i-2). The contour detection software similarly highlighted the radioluminescent signal distribution at the injection site in the atherosclerotic plaque (Fig. 4c:ii-3, iii-3). The 3D plots of the radioluminescent signal intensity was consistent with these results and revealed that the highest signal peak was associated with the ^18^F-FDG injection site in the plaque area (Fig. 4c:ii-4, iii-4); the control site was not above the noise level (Fig. 4c:i-4). A confirmatory image was obtained with an IVIS-200 (Fig. 4b) demonstrating a 62-fold brighter radioluminescent signal (3.06 × 10^5^ ± 3.03 × 10^4^ vs. 4.93 × 10^3^ ± 6.66 × 10^2^ p/sec/cm^2^/sr, p = 0.003) from the carotid endarterectomy samples compared to pre-injection controls (Fig. 4f). A linear relationship was observed in both CIRPI (Fig. 4e) and confirmatory IVIS-200 (Fig. 4g) images of all human samples during post-injection radioluminescent signal vs. multiple exposure times. Figure 4 is already published in the SNMMI Annual Conference Abstract^19^.

#### PAT Imaging

**Figure 5:**
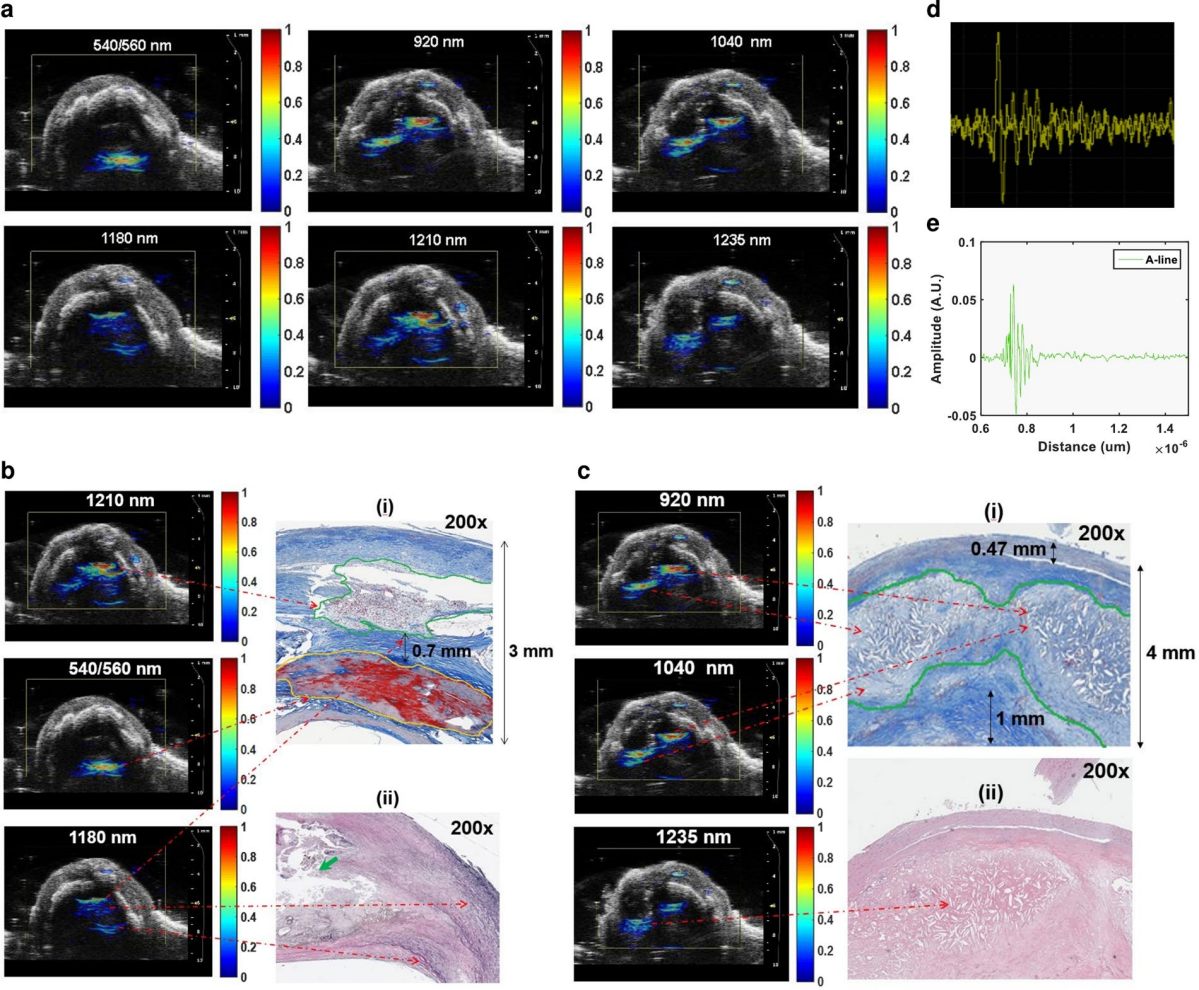
CIRPI images correlate with conventional ultrasound and histological images. Reconstructed PAT images at multiple wavelengths are superimposed on a Vevo 2100 collected ultrasound image of the same human carotid endarterectomy sample shown in Fig. 4. A precise alignment is observed between the conventional ultrasound and the PAT images. **(a)** Human atherosclerotic plaques show complex structure when laser is tuned at various wavelengths (540/560 nm, 920 nm, 1040 nm, 1180 nm, 1210 nm, and 1235 nm) to identify different plaque compositions. PAT images for 540/560, 1180, and 1210 nm wavelength belong to specimen 1; 920, 1040, and 1235 nm belong to specimen 2. **(b)** In specimen 1 PA signals are identified at (i) 540/560 nm, 1210 nm, and 1180 nm representing the presence of calcification (1 mm long), cholesterol (1 mm long), collagen/elastin (0. 7 mm thick), respectively. However, for specimen 2 PA signals are detected at (ii) 920 nm, 1040 nm, and 1235 nm yield the presence of cholesterol ester, phospholipid, and triglyceride in 2 mm area, respectively. **(b-i)** Histochemical analysis of transverse or axial plane of the specimen 1 (200×) with Trichrome stain highlights various compositions of the plaque that covers over 90% of the 3 mm thick vessel. The compositions of the plaque include 1.0 mm area of lipid and cholesterol (green outline) that matches with the corresponding PAT image for 1210 nm wavelength. Orange outline highlights a 1.0 mm mineralized calcium material correspond to zones of calcification in the PAT image for 540/560 nm wavelength. Blue stain highlights 0.7 mm collagen deposition that matches with the PAT image for 1180 nm wavelength. **(b-ii)** Histochemical analysis of specimen 1 (200×) with EVG stain highlights elastic fibers (black stain pointed with green arrow adjacent to the plaque lipid/cholesterol) that matches with the corresponding PAT image for 1180 nm wavelength. **(c-i)** Histochemical analysis of specimen 2 (200×) Trichrome stain outlines two prominent lobes of cholesterol and lipid deposition (green outline) measuring 2.5 mm in thickness corresponds to the PAT images at 920 nm (cholesterol ester) and 1040 nm (phospholipids) wavelengths. The normal luminal wall thickness is less than 0.5 mm thick with normal vessel wall material (double black arrow). Collagen deposition (dark blue staining material, double black arrow) measured 1 mm in thickness. **(c-ii)** Histological analysis of specimen 2 (200×) with H&E stain highlights giant cells with cholesterol cleft representing severe lipid and cholesterol corresponds to the PAT image for 1235 nm (triglyceride). **(d)** A single PA signal (A-line) captured with an oscilloscope; **(e)** an A-line is captured when the CIRPI probe is placed in close proximity of 0.8 µm of the human carotid atherosclerotic plaque. Figure 5 is already published in the SNMMI Annual Conference Abstract^19^.

The CRIPI PAT images of human carotid endarterectomy samples highlighted disease tissue compositions of atherosclerotic plaque─calcification (540/560 nm), lipid/fatty acid accumulation in the form of mostly cholesteryl ester (920 nm), phospholipids (1040 nm), cholesterol (1210 nm), and triglyceride (1235 nm). Also, we observed the presence of elastin/collagen at 1180 nm in PAT images (Fig. 5a). Although, we observed all these disease tissue constituents in most of the human carotid endarterectomy samples (n = 7), we only present here the quantitative analysis of two different human samples for simplicity. Images obtained at 540/560 nm, 1180 nm, and 1210 nm belonged to specimen 1 (Fig. 5b) whereas those at 920 nm, 1040 nm, and 1235 nm belonged to specimen 2 (Fig. 5c). At the inner lumen of the specimen 1, we observed a 1 mm area of severe calcification (540/560 nm) along with a 1 mm length of cholesterol (1210 nm) deposition close to the outer lumen (Fig. 5b-i). In between the cholesterol and calcium deposition, a 0.6–0.7 mm length of elastin/collagen (1180 nm) was also observed (Fig. 5b-i,b-ii). In the specimen 2, we identified two lobe shaped severe cholesterol ester (920 nm) depositions measuring 2.5 mm (right) and 1 mm (left) in thickness (Fig. 5c-i). These two lobes also consisted of higher phospholipids (higher PA signal, 1040 nm) compared to triglyceride (lower PA signal, 1235 nm) (Fig. 5c-ii). Approximately 1 mm area of elastin/collagen was also identified in close proximity to these lobes (not shown).

#### Histologic Analysis

**Figure 6:**
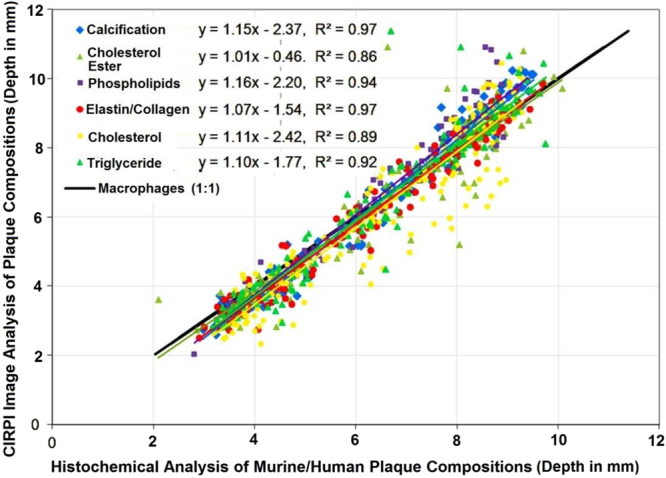
Correlation between CIRPI images and histochemical measurements of plaque compositions are computed using the Pearson product-moment correlation coefficient. A p-value of less than 0.01 was considered statistically significant. Each composition is identified based on their depth in mm. The Pearson correlation coefficients of calcification (R^2^ = 0.97, p < 10^−5^), cholesterol ester (R^2^ = 0.86, p < 10^−5^), phospholipids (R^2^ = 0.94, p < 10^−5^), elastin/collagen (R^2^ = 0.97, p < 10^−5^), cholesterol (R^2^ = 0.89, p < 10^−5^), triglyceride (R^2^ = 0.92, p < 10^−5^) illustrated a strong linear relationship between the CIRPI and the histochemical analysis. However, for the macrophages we found the strongest linear correlation (R^2^ = 1, p < 10^−5^).

Specimen 1 demonstrated atherosclerotic plaque measuring over 90% of the thickness of the vessel wall (Fig. 5b-i). The plaque measured 3.0 mm in thickness with a 1.0 mm thick area of mineralized/calcified material (red color area on Trichrome stain outlined with orange color), a 1.0 mm thick zone of lipid/cholesterol accumulation (outlined with green color), and 0.7 mm thick region of collagen depositions (dark blue staining material highlighted with black double arrow). In addition, we also observed frayed black staining elastic fibers adjacent to the lipid/cholesterol plaque on EVG stain (Fig. 5b-ii). In specimen 2, we identified 4.0 mm of wall thickness, where less than 0.5 mm belonged to the original vessel wall (black double arrow) and 3.5 mm represented the plaque thickness (Fig. 5c-ii-ii). This atherosclerotic plaque exhibited two lobe shaped prominent lipid and cholesterol (in form of cholesterol clefts) accumulation in a 2.5 mm thick area (Fig. 5c-i, outlined with green color). In this plaque, collagen deposition was measured at 1.0 mm in thickness (black double arrow). Figure 5 is already published in the SNMMI Annual Conference Abstract^19^. These quantitative histochemical analyses of human carotid endarterectomy samples were highly correlated to the data obtained through our CIRPI system (Fig. 6). The Pearson correlation coefficients of calcification (R^2^ = 0.97, p < 10^−5^), cholesterol ester (R^2^ = 0.86, p < 10^−5^), phospholipids (R^2^ = 0.94, p < 10^−5^), elastin/collagen (R^2^ = 0.97, p < 10^−5^), cholesterol (R^2^ = 0.89, p < 10^−5^), triglyceride (R^2^ = 0.92, p < 10^−5^) illustrated a strong linear relationship between the CIRPI and the histochemical analysis. Specimen 2 also showed the presence of moderate accumulation of macrophages, chronic inflammation, lipid, and some areas of calcification. For all human samples occlusions ranged between 20–55%. See Table 1 in *Supplementary Results* for details on histochemical analysis and CIRPI results of murine model.

## Discussion

Although, ^18^F-FDG has become an important metabolic contrast agent in atherosclerosis PET imaging, its lack of specificity obscures ^18^F-FDG signals from the artery of interest due to highly metabolically active myocardium. In this study, the intravascular imaging with the CIRPI system has overcome this limitation of ^18^F-FDG uptake by the myocardium. The CIRPI system can detect the inflammation at the ligation and its nearby arterial vessel lumen through detecting the macrophages accumulation that uptake large quantity of ^18^F-FDG. Thus, in this study, specificity of ^18^F-FDG signal is highly defined.

We spatially co-localized the macrophage accumulation in murine LCA based on visible photons detected with the scintillating imaging window of the CIRPI system during the ^18^F-FDG decay only at the ligation area and its close vicinity (extending 1 mm from the ligation). This mouse model results in macrophage-rich plaques within the luminal wall in close proximity to the ligation identified through histochemical analysis that also matched with the area where the radioluminescent signals were collected with our CIRPI system. We used the ligation (enlarged area) location as a landmark for spatial co-localization.

Using both human and murine carotid arterial plaques, we have demonstrated that our hybrid dual-modality catheter-based CIRPI system detects ^18^F-FDG uptake with high-resolution and high-sensitivity, and is capable of simultaneously characterizing plaque morphologic constituents. These findings were verified with standard optical and ultrasound/PA imaging systems, as well as autoradiography and immunohistochemical analyses of molecular composition and pathologic status.

Thus, our integrated CIRPI system offers an unprecedented opportunity for intravascular detection and characterization of the biology and morphology of atherosclerotic plaques that provides a multiparametric analysis of pathognomonic constituents of vulnerable plaques. This approach can both broaden our understanding about human CAD and enable prognostic data for this deadly disease. Such measurements have important implications for guiding treatment to reduce the risk associated to heart attack and its effect on the society.

A limitation of this study is that the probe diameter is larger than the murine carotid artery. Thus, we performed *ex vivo* murine experiments. Due to the small size of the murine carotid artery, the entire length of the artery was imaged longitudinally. We were able to detect the locations of the macrophage accumulations and the compositions of elastic/collagen based on the detected radioluminescent and photoacoustic signals from specific areas of the plaques. Our murine model, which combines risk factors of hyperglycemia and hyperlipidemia, was developed and studied extensively to provide a complex inflammatory plaque^20,22^. However, as with most other animal models, this model does not show thin fibrous caps or rupture/thrombosis typical of vulnerable human lesions. The excised human carotid plaques from symptomatic patients were studied to provide this complementary information. Simultaneously, the histochemical analysis was performed and independently verified.

Another limitation of this study is the use of local ^18^F-FDG injection in the *ex vivo* human carotid endarterectomy samples rather than systemic intravenous injection, which would have had to occur pre-operatively. The excised human plaques from patients with symptomatic carotid stenosis (the primary reason for surgery) provided a variety of plaque tissue components for testing our CIRPI system. We used a very low dose (50 µCi) of ^18^F-FDG to demonstrate detection sensitivity. As complementary data, the murine model did include intravenous injection of ^18^F-FDG and showed plaque uptake and CIRPI detection. A larger human study, including differentiating plaques from symptomatic vs. asymptomatic carotid stenosis, should be considered in future testing our CIRPI system.

A key next step in translating this technology toward human application will be further miniaturization of the CIRPI system to coronary size (<3 mm) and intravascular testing *in vivo*. We have prior experience with rabbit atherosclerosis models, where the aorta is similar in size to the human coronary artery. The intravascular CIRPI system will be validated with autoradiography, OCT, histology, and immunohistochemistry analyses.

Unlike external imaging, γ and x-ray radiation is not desirable for intravascular detection due to their deep penetrating ability and overwhelming background noise from outside the vessel (e.g., in the myocardium)^24^. Thus, it is difficult to see the relatively weak signal from the vulnerable plaques. Therefore, β-radiation from ^18^F-FDG was chosen in this study for their limited penetration depth of only a few millimeters and their radiation-sensitivity towards CaF_2_:Eu based CIRPI probe. The CaF_2_:Eu based scintillating imaging window was specially designed to detect and localize lesions depending on both the physical properties and the biologic behavior of ^18^F-FDG.

Annihilation photons cause intrinsic efficiency of 10% when scintillator thickness is approximately 1.0 mm. Therefore, we chose to use a scintillating imaging window with 0.125 mm thickness to eliminate a significant amount of annihilation photons coming from distant tissues, causing to hinder imaging of the local ^18^F-FDG uptake in the plaque. Although, 300-keV charged β-particles are likely to deposit all of its energy on our thin scintillator based imaging window, γ-particles may deposit a very small fraction of its energy due to Compton interaction. However, we were not able to detect any background noise produced by these γ-particles. This observation leads to no subtraction of γ-radiation signal. In addition, due to geometric sensitivity of the radiation-sensitive catheter-based probe design and structure of atheroma, only particles discharged towards vessel lumen will be detected, unlike basement membrane (traveling away from the scintillator).

We used a 0.2 mCi of ^18^F-FDG dose in this murine feasibility study^25^ and much less (0.05 mCi) for human carotid endarterectomy samples. A typical recommended ^18^F-FDG dose for an adult human is between 10–20 mCi. Therefore, in our murine model we used approximately 400× less ^18^F-FDG dose (50 µCi) compared to recommended human dose, when adjusted for weight^26^. We were encouraged that >60-fold higher ^18^F-FDG signals were detected from the arterial plaques of both human and murine samples compared to controls. This 60-times higher radioluminescent signal from the *ex vivo* murine models compared to our 2015 study (4-fold) is not due to partial volume effect rather it is due to two factors (1) new optical design of the imaging system (2) biological variability. We completely redesigned the optical components by implementing a miniature microscope to enhance the signal to noise ratio (SNR). The signal collecting probe geometry is much different than the balloon-enabled scintillating probe deigned in 2015. We used a green lens in the front of the 18,000 optical imaging fibers bundle that has 7.4 µm of pixel size. Although, the imaging fiber bundle had 18,000 optical fibers in the probe in 2015 study, the pixel size was 11.6 µm. Thus, finer details can be captured with the current optical fiber bundle. Also, we have used ray tracing software called Zemax to model the worst- and best-case scenarios of each optical path for various Grin lens which was not done for the study published in 2015. With respect to biological variability, it is possible that the number of macrophages are much higher than the one we have imaged previously. In this study, we have observed large quantity of macrophages that fill up the entire lumen; however, in our previous study we observed necrotic core, an advance stage in the atherosclerotic plaque, which indicate a healing process with less macrophage accumulation. It is also possible that the macrophages will have up taken much higher amount of ^18^F-FDG due to heterogeneous uptake pattern.

The central hypothesis of this study was to detect and characterize vulnerable plaques using our CIRPI system. PAT imaging can provide sufficiently well differentiated absorption spectra from calcification and lipids compared to other compositions of normal arterial tissue between 740–1400 nm wavelength due to their thermo-elastic expansion. Similar to most conventional PAT, our PAT image reconstruction methods are quantitatively based using a two-dimensional numerical finite-element method. Our CIRPI system images only the distribution of absorbed light energy density, which is generated from local optical absorption coefficient of the disease tissue and optical fluence distribution within the irradiated medium. It has been known that the absorption coefficient of tissue is directly correlated with tissue structure and tissue compositions such as calcification, cholesterol, and triglyceride. For example in Fig. 5(b), in specimen 1, we observed higher signals at 540/560 nm and 1210 nm wavelength compared to 1180 nm. Due to the direct correlation between the signal intensity and the severity of the disease composition we concluded that this particular human had severe calcification and cholesterol components compared to elastin/collagen. Based on histochemical analyses of specimen 1, atherosclerotic plaque measured 3.0 mm in thickness with a 1.0 mm thick area of mineralized/calcified material, a 1.0 mm thick zone of lipid/cholesterol accumulation, and a 0.7 mm thick region of collagen depositions. In addition, we also observed frayed black staining elastic fibers adjacent to the lipid/cholesterol plaque on EVG stain.

We observed calcification, cholesterol ester, phospholipid, cholesterol, and to a lesser extent triglyceride in all human carotid endarterectomy samples with our CIRPI system, and confirmed with the quantitative map of histochemical analysis. The human tissue histochemical analysis were conducted independently by a trained pathologist. Special software was used for histochemical analysis to spatially quantify the tissue thickness, shape, and size of the plaque area and their compositions. We compared our quantitative PAT findings to the histochemical analyses of these same human tissue samples using the Pearson product-moment correlation coefficient. We found correlation coefficient values of calcification, phospholipids, triglyceride, cholesterol, cholesterol ester, and elastin/collagen ranges from R^2^ = 0.86 to R^2^ = 0.97 (p < 10^−5^), indicating a strong correlation between our PAT and histologic findings respect to the disease compositions, their severity, locations, and shapes. The localized murine plaques, by contrast, demonstrate inflammation (detected by ^18^F-FDG) but not the substantial and complex lipid/fatty acid +/- calcification seen in advanced human plaques. Thus, the only significant PA signal seen was from the ubiquitous elastin/collagen in the vessel wall, as confirmed by histology. Further, a one-to-one correlation was observed between our CIRPI and histologic findings respect to elastin/collagen (R^2^ = 0.97, p < 10^−5^) and macrophages (R^2^ = 1, p < 10^−5^) with CIRPI. An important note on spatial co-localization is that it was an important aspect of this study that the PAT images from our CIRPI system can be correctly oriented with respect to the US images of the disease tissue.

The ultimate value of improving diagnosis is to guide treatment to positively impact patient care and outcomes. Current plaque imaging primarily provides information about plaque size and lumen encroachment, while our CIRPI system can provide both biological status and detailed structural information about plaques─key variables in assessing plaque vulnerability. Thus, plaques found to have high-risk features may benefit from intensive pharmacological (e.g., PCSK9 inhibitors) and mechanical (e.g., stenting) interventions to prevent near-term clinical events, while low-risk plaques can be treated with more conventional preventive therapy (i.e., statin, aspirin, diet, exercise). Changing the future of how CAD is diagnosed and managed clearly requires much more extensive testing and validation of the technology itself, as well as outcome and intervention trials to show benefit in the care of this devastating disease.

## Conclusions

Our multimodality molecular and tissue imaging approach comprised of an integrated fiber-optic CIRPI system, harnessing radioluminescence and sound to both detect and characterize vulnerable atherosclerotic plaques. We were able to outline the plaque location with characteristic information pertaining to specific tissue constituents. Our catheter-based system provides the high sensitivity and resolution needed for coronary plaque imaging, plus radioluminescence and photoacoustic imaging are ripe for clinical translation, particularly as there are multiple FDA-approved radionuclides (e.g., ^18^F-FDG). We demonstrated the potential for clinical detection and evaluation of vulnerable human coronary atherosclerosis via CIRPI, plus the potential for expanding our understanding of CAD and ultimately improving how we manage this disease.

Note: The data presented in this manuscript in the form of Figs 1–5 have been already published in the Society of Nuclear Medicine and Molecular Imaging (SNMMI) Annual Meeting Abstract, May 2017^19^.

## Electronic supplementary material

Supplemental File
