# Supplementary material for: A Dual-Modality Hybrid Imaging System Harnesses Radioluminescence and Sound to Reveal Molecular Pathology of Atherosclerotic Plaques
Source: Sci Rep. 2018 Jun 12;8:8992. doi: 10.1038/s41598-018-26696-8 (PMC5997702; doi:10.1038/s41598-018-26696-8)
Supplement: Supplementary file 1 — Supplemental File [file 41598_2018_26696_MOESM1_ESM.pdf]

A Dual-Modality Hybrid Imaging System Harnesses Radioluminescence and Sound to Reveal Molecular Pathology of Atherosclerotic Plaques

Raiyan T. Zaman[1,7]*, Siavash Yousefi[2], Steven R. Long[3], Toshinobu Saito[1], Michael Mandella[4], Zhen Qiu[5], Ruimin Chen[6], Christopher H. Contag[4,5,7,8,9], Sam Gambhir[5,7,9], Frederick T. Chin[5,7], Butras T. Khuri-Yakub[10], Michael V. McConnell[1,7], K. Kirk Shung[6], Lei Xing[2,7]

1. Division of Cardiovascular Medicine, Department of Medicine, Stanford University School of Medicine
2. Division of Medical Physics, Department of Radiation Oncology, Stanford University School of Medicine
3. Department of Pathology, Stanford University School of Medicine
4. Department of Pediatrics (Neonatology), Stanford University School of Medicine
5. Department of Radiology, Stanford University School of Medicine
6. Department of Biomedical Engineering, Viterbi School of Engineering, University of Southern California
7. Molecular Imaging Program at Stanford University (MIPS), Stanford University School of Medicine
8. Department of Microbiology and Immunology, Stanford University School of Medicine
9. Department of Bioengineering, Stanford University Schools of Medicine and of Engineering
10. Department of Electrical Engineering, Stanford University

SUPPLEMENTARY MATERIALS AND METHODS

Circumferential Intravascular Radioluminescence-Photoacoustic Imaging (CIRPI)

Circumferential Radioluminescence Imaging (CRI) Peripheral System Design. The optical components of the CIRPI system consists of a 10x magnification infinity-corrected microscope objective (RMS10x, Olympus Inc.) with a working distance of 10.6 mm, numerical aperture (NA) of 0.25 and effective focal length (EFL) of 18 mm (Fig. 1a). The system also contains an infinity-corrected tube lens for plan fluorite objective (ITL200, Thorlabs) in between the objective (F2 = 102 mm) and the ProEM charge-coupled device (CCD) camera (F3 = 200 mm) (Princeton Instruments). The CIRPI system provides a 360˚ view inside an artery through an innovative probe design (see section on Dual-Modality Endoscopic Probe).

The novelty of this CIRPI system is based on a scintillating window made from organic calcium fluoride doped with europium (CaF_2:Eu) phosphor, placed on the catheter-based probe to convert the β-particles of the positron emission signal into visible light due to radioactive decay (primary decay time 940 ns). The molecules in the scintillating window get excited with the incoming ionizing radiation, especially β-particles from the decay of ^{18}F-FDG, or other isotopes, by absorbing energy. When these molecules return to a lower energy state from an excited state, they release optical radiation in the visible range. This light is then captured with a *highly sensitive* CCD camera using deep thermoelectric cooling at -70 °C for minimizing the background signal from the temperature-dependent dark current, hot pixel blemishes, and vibration. The deep thermoelectric cooling was done with a CoolCUBE II liquid circulatory system. The

camera exposure times were set to 1, 5, 10, 15, 30, 45, 50, and 60 seconds. However, the best sensitivity was observed at 45 second exposure times (above this threshold the pixels were saturated). For ^{18}F-FDG imaging with the CIRPI system, the binning factor was set at smallest limit (1x1 pixel) and also a medium setting (4x4 pixels) was used, with effective active imaging resolutions of 1024 x 1024 pixels and 128 x 128 pixels (width x height), respectively. The analog-to-digital conversion speed was set to 10 MHz with corresponding EM gain of 50 at 16-bits bit depth. The storage shift rate was set to 600 ns with readout time of 4 ms for ‘frame transfer’ readout mode. The CCD camera shutter mode was set to “always open” without any delay (0 ms) in opening and closing. The selection process of optimal scintillating material and resolution were described in the previously published manuscript [1].

Scintillating Window. The scintillating window was fabricated from a polymer based polyvinyl toluene block made of CaF_2:Eu phosphor (Saint-Gobain Crystals, Hiram, OH, USA). CaF_2:Eu was selected as the optimal scintillating materials with respect to highest radioluminescent signal[1]. The phosphor has a refractive index of 1.58 with 68% light output. The scintillating efficiency was 10,400 photons/1 MeV e-. It has a maximum emission at 435 nm wavelength with a temporal pulse width of 2.2 ns (FWHM). The scintillating window was machined into a cylindrically shaped tube with 10 mm length and wall thickness of 0.125 mm (Figs. 1b-1d). The outer diameter (OD) and inner diameter (ID) of the window is 3.75 mm and 3.5 mm, respectively. Radioluminescence is produced within the scintillating imaging window following the emission of a beta particle from a radiotracer (^{18}F-FDG) within a macrophage. The optical photons were captured by a high-

numerical-aperture 10x microscope objective coupled to a deep-cooled ProEM CCD camera. This scintillating window can detect radioluminescent signal within the travel distance of beta-particles from the decay of the ^{18}F-FDG (2-3 mm).

Dual-Modality Endoscopic Probe. The CIRPI probe has a 840 mm flexible tether with up to 17˚ of bending freedom and a 41 mm long rigid distal imaging head consists of (1) a CaF_2:Eu scintillating imaging window, (2) a light guiding multi-mode optical fiber (OF-1, 0.22 NA, 0.2 mm core diameter; Thorlabs Inc., USA), (3) a leached image fiber (OF-2, 18,000 optical fibers represent a 0.9 mm diameter imaging area with 7.4 µm pixel size; Schott Inc., USA), (4) a single-element ultrasonic transducer (Lithium Niobate LNO, 40 MHz, unfocused, ring transducer with OD = 3.0988 mm, ID = 1.37 mm, and length of 5.1 mm), (5) a digital actuator (2.0 mm diameter, 18.62 mm long; Namiki Precision Jewel Co., Ltd. Japan), and (6) 45˚ degree flat rotating mirror (3 mm diameter, protected aluminum on glass substrate, with the reflection surface at 45° to the probe's axis; Edmund Optics Inc., USA) that was placed in a stainless steel tube housing (Fig. 1e). A single-element ultrasonic transducer was used for achieving a high Signal to Noise Ratio (SNR) at MHz repetition rates. Laser pulses from a portable UV-VIS-NIR tunable laser (7 ns pulse length at 20 Hz repetition rate) were coupled into a multimode fiber using a 10x microscope objective. These laser pulses are guided by the multi-mode optical fiber which is also positioned parallel to an imaging fiber-bundle belonging to the CIRPI system. These pulses are then emitted into the tissue through a central hole (1.37 mm diameter) in the transducer located in the distal end of the probe. The distal end of the OF-2 was terminated by a 1 mm diameter Gradient-Index (GRIN) lens designed to have a working

distance of 5 mm, and paraxial magnification of 5.86 (Fig. 1f). The GRIN lens provides an NA of 0.5, and radial index gradient has a maximum central refractive index of 1.635 at the lens axis.

Circumferential sector scanning (B-scan) was accomplished by rotating a 45˚ degree flat mirror. The mirror was driven by a geared actuator (gear ratio, 254:1) to steer the laser beam from the optical fiber to the tissue and the acoustic wave from the tissue to the ultrasonic transducer (UST). The detection speed for a 360˚ degree view of an artery took only few minutes (≈4 minutes). When the pulsed laser light illuminated atherosclerotic plaque, the optical absorbers there (such as lipid, cholesterol, calcification) undergo thermo elastic expansion, generating an acoustic pressure wave which was detected with the UST. The mirror was also important for reflecting visible light to the OF-2 due to the vibration of molecules within the scintillating imaging window upon the deposition of β-particles energy from the ^{18}F-FDG decay. As water and glass have a large ratio of sound propagation speeds, the scanning mirror has exhibited total external reflection within the acceptance angles of the ultrasonic transducer and the GRIN lens, and thus, contributed to no additional propagation losses into the ultrasonic and visible light detection. The scanning mirror system had enabled circumferential B-scanning without moving other illumination optics and the ultrasonic sensor. The optical fibers, the transducer's signal wires, and the actuator wires were encapsulated in a flexible catheter body with outer diameter of 3.2 mm where 0.5 mm was used for a catheter enclosure. The mirror's rotational speed was kept constant, while providing a matching medium for acoustic wave propagation, the transducer and scanning mirror's housing space was water-coupled with deionized water through a 0.25 mm hole on the 0.125 mm thick

scintillating imaging window. The actuator was isolated from the water with a spacer, and the torque was required for the mirror rotation that is transferred through a micro-magnetic (OD 1.58 mm with a length of 3.175 mm, K&J Magnets Inc., USA) coupling mechanism.

***Photoacoustic Tomography (PAT) Peripheral System Design*.** PAT images are created when tissues are excited with pulse laser irradiation to emit ultrasonic waves that can be reconstructed similarly to US images, but representing a spatial map of optical absorption by endogenous constituents (e.g., calcification, cholesterol) or exogenous contrast agents. Optical absorption is wavelength dependent, therefore multiple wavelengths imaging allows “spectral unmixing” of the generated PA signals based from measured optical absorption spectra of calcification and other photo absorbers. Therefore, information about the relative concentrations of endogenous chromophores or exogenous contrast agents can be determined allowing enhanced signal to background ratios. The low optical absorption and US scattering of disease tissues makes it optimal for PAT imaging at high resolution and depth (3-5 cm deep).

For PAT, we used a portable tunable laser (410-2400 nm Opolette 355 LD, Opotek Inc. Carlsbad, CA, USA) with 3-4 mm beam diameter (before focusing into the 200 µm core multimode fiber). The peak wavelength of the laser’s spectral bandwidth can be tuned to multiple values for selective imaging of the different tissue constituents. The central hypothesis of this PAT imaging is that the absorption spectra of calcium, lipids, elastic, and collagen are sufficiently well differentiated from those of the constituents of normal arterial tissue in the 500 to 1400 nm wavelength range to permit the detection of calcium or lipid-rich plaques using PAT imaging. The experiments described herein used

laser tunings of 540-560 nm (calcification), 920 nm (cholesteryl ester), 1040 nm (phospholipids), 1180 nm (elastin/collagen), 1210 nm (cholesterol), and 1235 nm (triglyceride).

The repetition rate was set to the highest at 20 Hz with 7ns FWHM pulse length. The laser was linearly polarized at <2 mrad divergence. The laser was connected to ICE450 pump laser power supply box for power and coolant circulation. The power supply box and laser were both controlled through the Opelette Control Box. A pulse signal generator (SDG 5082, 80 MHz, 500MSa/s, Siglent Technologies America Inc., USA) provides the flash lamp trigger to the laser with an internal Q-switch delay of 140 µs at 200 µs FWHM with a 20 ns rise time and peak voltage of 5 Vpp (min). The laser then sent the Q-switch triggering output to a 4-channel digital delay/pulse generator (DG535, Stanford Research Systems, USA). The total delay between Q-switch and flash light was 640 µs where the flash lamp triggering input delay was 500 µs. A 200 MHz computer controlled pulser-receiver (5900, Panametrics-NDT, Olympus Inc., USA) was used to trigger a two channel digital storage oscilloscope (SDS2202X, Siglent Technologies America Inc., USA) and receive the PA signal. The PA signals were read from the oscilloscope by EasyScopeX software (Siglent Technologies America Inc., USA) and stored in a laptop computer. Each single A-line (Fig. 3b) was acquired every 1.42° angular rotation at a 0.73 µm distance between the probe and the tissue (Fig. 3c). Based on 330 A-lines a single B-scan was created. A 360˚ degree view of an artery was implemented by 66 B-scans. A 1.42° angular step between two successive A-lines in a B-scan image was determined by the gear ratio (254:1) of the actuator used in the novel miniaturized imaging probe described below.

Implementation of Edge Detection Software. An edge detection method was implemented for finding object boundaries in radioluminescence images. We used Sobel edge detection algorithm for identifying discontinuities in brightness. We considered object to be segmented that differs greatly in contrast from the background image. The changes in contrast was detected by operators that calculated the gradient of an image. The gradient image was calculated by applying a threshold to create a binary mask containing the segmented cell. Sobel operator was used to calculate the threshold value. We then tuned the threshold value to 0.5 for obtaining a binary gradient mask that contains the segmented cell. After the dilation of this mask, the binary gradient mask showed lines of high contrast in the image and when the Sobel image was dilated using linear structuring elements followed by the horizontal structuring element outlined the area with macrophages. However, these images were reconstructed with filled holes followed by removing diagonal connectivity at the region with higher radioluminescence signal. Finally, the segmented object was smoothed by eroding the image twice with a diamond structuring element. The entire process takes 10 seconds to perform. This technique is not based on region of interest. Whenever the software detected any edge it highlighted the area after the entire computation was done.

Verification Imaging

Maestro fluorescence imaging system. Maestro *in-vivo* imaging system (CRi, Woburn, MA) is a high performance multi- and hyper-spectral imaging system designed for fluorescence macro-imaging. The system uses an Imaging Module and an Illumination Module for fast and accurate imaging and a liquid crystal element that enables the

transmitted light to be electronically tuned. We used this system for a macroscopic image of the murine tissue samples.

External Optical Imaging System (IVIS-200). The experimental procedure using IVIS-200 imaging has been previously described[2]. In brief, the IVIS-200 system (Xenogen Product of PerkinElmer Inc., Waltham, MA, USA) yields for bioluminescence and fluorescence optic-based imaging and was used to validate the CIRPI system. Although, IVIS-200 imaging is designed to provide bioluminescence signal, an addition of a scintillator screen enables a radioluminescent signal detection. Samples were placed inside the light-tight imaging chamber and the field-of-view set to 1.5 cm for ^{18}F-FDG enriched samples, with the highly sensitive CCD camera system cooled to -90 °C. For ^{18}F-FDG imaging, the binning factor was set at medium (8x8 pixels) and an LSO scintillator screen was placed on top of the tissue sample. The bioluminescence mode was used to image the radioluminescent from the β-particles of the positron emission signal from ^{18}F-FDG radioactive decay, with the exposure time of 1, 10, 15, 30, 45, 50, and 60 seconds. An average radiance was calculated with a unit of p/sec/cm^2/sr based on the IVIS-200 images after correction for field flatness.

Vevo LAZR/ Vevo 2100 Imaging System. The Vevo LAZR system was used to verify our PAT results with our CIRPI system. This system incorporated photoacoustic imaging into high-resolution ultrasound (US). The laser for this system was transmitted at 40 MHz repetition rate with 100% energy efficiency. For the acquisition the PA gain was set to 40 dB with 2D gain of 22 dB. The depth and width for the imaging was set to 10 mm and

14.08 mm, respectively. We also perform verification experiment with Vevo 2100, a high-frequency, high-resolution digital imaging platform with linear array technology for 3D ultrasound imaging. Each frame in 2D was collected up to 740 frame per second (fps).

Autoradiography. The autoradiography procedure used for validation has been previously described [2]. In brief, autoradiography was used to directly image the radiation emitted from the ^{18}F-FDG tissue samples with high spatial resolution [3,4] for further validation of the radioluminescence images with our CIRPI system. Carotid samples were placed in contact with a super resolution storage-phosphor screen (12.5×25.2 cm, PerkinElmer Inc., MA, USA). Over a sufficient exposure time (48 hours), emitted β-particles were recorded as an image on the storage-phosphor screen. The film was then read out by a Cyclone reader using a spatial resolution of 600 dpi (PerkinElmer Inc., Waltham, MA, USA). Using these techniques, the spatial distribution of radioisotope uptake of the tissue sample was recorded as digital light units (DLU) per mm^{-2}.

Co-registered with Histological Analyses. After the experiments, both the murine (n=6) and human (n=7) samples were place in 10% formalin, and submitted for paraffin embedding, sectioning, and staining. Step sections (5 µm thick, n=16) were collected at different levels beginning at one end of the sample and proceeding to the other end. The sections were mounted on glass microscopic slides, stained with hemotoxylin and eosin, trichrome (collagen), and EVG (elastin) reagents and covered with Tissue Tek Film as cover slips.

The microscopic slides were examined with an Olympus BX51 microscope fitted with diffuse white light (LM) and transmission polarizing (TPM) optics and 2x, 4x, 10x, 20x, and 40x objectives. Selected fields were imaged by pathologist at Stanford SOM using Zeiss Axiocam MRc5 digital camera mounted on the microscope and Zeiss AxioVision SE64 Rel 4.8 software supported by a Dell Optiplex 980 Desktop Computer. The histological study was based on a quantitative analysis of disease tissue constituents that were co-register with the CIRPI results.

Safety Assurance. After each experiment, we used classical gamma counting analog meter, Geiger-Muller counter, to perform safety assurance on the instruments, counter top, whole body, and lab coat, that there was no contamination from ^{18}F-FDG. Each time the count per minute was around 30-50 CPM that was in normal range of 0.03-0.05 mR/hr.

Statistical Analysis

A pairwise two-sample Student's t-test was performed to compare ex vivo signal intensity from the ^{18}F-FDG-enriched murine ligated LCA and non-ligated (control) RCA (n=10). The same t-test was performed for human carotid plaques for pre and post ^{18}F-FDG injection. The underlying distribution was found to be normally distributed according to QQ-plots for both murine and human samples. As murine had similar weight (25±0.006g) and same dose, these factors were not considered in the statistical analysis. For human samples, we injected the same dose to all equally cut with similar weight samples (0.2±0.001mg). Therefore, weight and dose were eliminated during the statistical

process. Murine and human samples were not randomized. Ex vivo analyses were performed using MATLAB software. We presented all values as mean ± standard deviation. We considered $P<0.05$ as statistically significant for all ex vivo analyses.

For a significance level of $P<0.05$ and a statistical power of 0.90, each group should comprise of at least N=6 animals to obtain a statistically significant result. We used an equation for sample size calculation for a study comparing two means: $N=\{4*\sigma^2(Z_{crit}+Z_{pwr})^2\}/ D^2$ where N is sample size, D is the minimum expected CNR difference based on the means of the two groups (plaque vs. control), σ is assumed standard deviation of each group (assumed to be equal for both group) be 1.65, $Z_{crit} = 1.960$ for significance level of 0.05(95) (standard normal deviate corresponding to selected significance criteria) and $Z_{pwr} = 1.645$ for statistical power of 0.95 (standard normal deviate corresponding to selected statistical powers) .

Correlation between the CIRPI images and histochemical measurements of plaque compositions are computed using the Pearson product-moment correlation coefficient. A p-value of less than 0.01 was considered statistically significant. Each composition is identified based on their depth in mm. The Pearson correlation coefficients of calcification ($R^2=0.97$, $p<10^{-5}$), cholesterol ester ($R^2=0.86$, $p<10^{-5}$), phospholipids ($R^2=0.94$, $p<10^{-5}$), elastin/collagen ($R^2=0.97$, $p<10^{-5}$), cholesterol ($R^2=0.89$, $p<10^{-5}$), triglyceride ($R^2=0.92$, $p<10^{-5}$) illustrated a strong linear relationship between the CIRPI and the histochemical analysis. However, for the macrophages we found the strongest linear correlation ($R^2=1$, $p<10^{-5}$).

SUPPLEMENTARY RESULTS

Table 1. Observations of Murine and Human Samples with Histochemical Analysis and CIRPI System

Species	Observation	
**Mice**	**Histochemical Analysis**	**CIRPI Image Analysis**
Gender: Male<br>Age: 8 weeks old FVB<br><br>all 10 animals show same results	1. 10% or less occlusion<br>2. Area of dilatation near the ligated end showed macrophage accumulation and thrombus<br>3. No mineralized calcium<br>4. No lipid/cholesterol<br>5. Small amounts of collagen deposition and presence of elastic fiber	1. Moderate macrophages<br>2. No calcification<br>3. No cholesterol ester, phospholipids, cholesterol, and triglyceride<br>4. Presence of elastin/collagen
**Human**		
Gender: Male<br>Age: 82 years	1. 30% lumen occlusion<br>2. Moderate macrophages<br>3. Moderate mineralized calcium<br>4. Severe cholesterol and lipid deposition with cholesterol cleft<br>5. Moderate inflammation<br>6. Presence of elastin/collagen	1. Moderate macrophages<br>2. Moderate calcification<br>3. Severe cholesterol ester, phospholipids, cholesterol, and triglyceride<br>4. Presence of elastin/collagen
Gender: Female<br>Age: 79 years	1. 20% lumen occlusion<br>2. Sparse macrophages<br>3. Severe mineralized calcium<br>4. Minimal inflammation<br>5. Severe cholesterol and lipid deposition<br>6. Presence of elastin/collagen	1. Sparse macrophages<br>2. Severe calcification<br>3. Severe cholesterol ester, phospholipids, cholesterol, and triglyceride<br>4. Presence of elastin/collagen
Gender: Male<br>Age: 63 years	1. 55% lumen occlusion<br>2. Sparse macrophages<br>3. Severe mineralized calcium<br>4. Minimal/Mild inflammation<br>5. Mild cholesterol and lipid deposition	1. Sparse macrophages<br>2. Severe calcification<br>3. Mild cholesterol ester, phospholipids, cholesterol, and triglyceride<br>4. Presence of elastin/collagen
Gender: Male<br>Age: 82 years	1. 55% lumen occlusion<br>2. Sparse macrophages<br>3. Moderate mineralized calcium<br>4. No cholesterol and lipid deposition	1. Sparse macrophages<br>2. Moderate calcification<br>3. No cholesterol ester, phospholipids, cholesterol, and triglyceride<br>4. Presence of elastin/collagen
Gender: Male<br>Age: 78 years	1. 30% lumen occlusion<br>2. Sparse macrophages<br>3. Severe mineralized calcium<br>4. No cholesterol and lipid deposition	1. Sparse macrophages<br>2. Severe calcification<br>3. No cholesterol ester, phospholipids, cholesterol, and triglyceride<br>4. Presence of elastin/collagen
Gender: Male<br>Age: 71 years	1. 25% lumen occlusion<br>2. Moderate macrophages<br>3. Moderate mineralized calcium<br>4. Severe lipid/giant cells with moderate cholesterol formation<br>5. Moderate chronic inflammation	1. Moderate macrophages<br>2. Moderate calcification<br>3. Severe cholesterol ester, phospholipids, cholesterol, and triglyceride<br>4. Presence of elastin/collagen
Gender: Male<br>Age: 63 years	1. 45% lumen occlusion<br>2. Sparse macrophages<br>3. Severe mineralized calcium	1. Sparse macrophages<br>2. Severe calcification

	4. Plaque is mixed of lipid and other materials	3. Severe cholesterol ester, phospholipids, cholesterol, and triglyceride<br>4. Presence of elastin/collagen

REFERENCES

1 Zaman, R. T. *et al.* Scintillating balloon-enabled fiber-optic system for radionuclide imaging of atherosclerotic plaques. *J Nucl Med* **56**, 771-777, doi:10.2967/jnumed.114.153239 (2015).

2 Zaman, R. T. *et al.* Fiber-Optic System for Dual-Modality Imaging of Glucose Probes 18F-FDG and 6-NBDG in Atherosclerotic Plaques. *Plos One* **9**, e108108, doi:10.1371/journal.pone.0108108 (2014).

3 Shikhaliev, P. M. *et al.* Positron autoradiography for intravascular imaging: feasibility evaluation. *Phys Med Biol* **51**, 963-979, doi:10.1088/0031-9155/51/4/014 (2006).

4 Ott RJ, M. J., Wells K The performance of a CCD digital autoradiography imaging system. *Phys Med Biol* **45**, 2011–2027 (2000).
